# Pulmonary function impairment patterns and their clinical correlates in patients with pulmonary tuberculosis complicated by pulmonary infection: a single-center retrospective cross-sectional study

**DOI:** 10.3389/fmed.2026.1870280

**Published:** 2026-07-08

**Authors:** Xin Li, Jinyan Wang, Jian Wang, Jili Yang, Yan Li, Yawen Li, Bofei Liu

**Affiliations:** 1 General Hospital of Ningxia Medical University, Yinchuan, China; 2 The Fourth People's Hospital of Ningxia Hui Autonomous Region, Yinchuan, China

**Keywords:** diffusion impairment, pulmonary function, pulmonary infection, pulmonary tuberculosis, respiratory reserve

## Abstract

**Background and Objective:**

Pulmonary tuberculosis is a major cause of respiratory morbidity worldwide, and pulmonary function impairment is increasingly recognized as an important consequence of the disease. However, the functional patterns of pulmonary impairment in patients with pulmonary tuberculosis complicated by pulmonary infection, especially in hospitalized populations, remain insufficiently characterized. This study aimed to describe pulmonary function impairment patterns in such patients and to explore their clinical correlates.

**Methods:**

This single-center retrospective cross-sectional study included hospitalized patients with pulmonary tuberculosis complicated by pulmonary infection who were admitted between November 2024 and October 2025, underwent bronchoalveolar lavage fluid metagenomic next-generation sequencing, and had interpretable pulmonary function results. The analysis was retrospective because all study variables were extracted from pre-existing hospitalization records and database entries rather than being prospectively collected for the present pulmonary function study. Although the parent project is an ongoing prospective study with follow-up, no longitudinal follow-up data were used in the present analysis. Clinical, laboratory, immunologic, gas-exchange, microbiological, and pulmonary function data were extracted from the institutional database and electronic medical records.

**Results:**

A total of 72 patients were included. Pulmonary function abnormalities were common. Diffusion impairment was the most frequent phenotype, occurring in 37 patients (51.4%), followed by reduced respiratory reserve in 36 (50.0%) and obstructive ventilatory impairment in 30 (41.7%). Restrictive ventilatory impairment, mixed ventilatory impairment, and small airway dysfunction were less common. Patients with diffusion impairment were more likely to be male than those without diffusion impairment (73.0% vs. 42.9%, *P* = 0.019), and smoking history showed a borderline between-group difference. Patients with reduced respiratory reserve had significantly higher IL-6 levels than those without reduced respiratory reserve [13.18 (5.52–38.00) vs. 4.53 (1.94–12.28) pg/ml, *P* = 0.005]. In exploratory multivariable analysis, no variable was independently associated with diffusion impairment or reduced respiratory reserve after adjustment, although lymphocyte count showed a non-significant positive trend for diffusion impairment.

**Conclusion:**

Pulmonary function impairment was highly prevalent in patients with pulmonary tuberculosis complicated by pulmonary infection, with diffusion impairment and reduced respiratory reserve as the predominant phenotypes. These findings suggest that pulmonary dysfunction in this population extends beyond conventional ventilatory defects and may involve substantial abnormalities in gas transfer and respiratory capacity. Comprehensive pulmonary function assessment may provide clinically relevant information for functional evaluation and individualized management in this patient group.

## Introduction

Tuberculosis (TB) remains one of the most important infectious diseases worldwide and continues to impose a substantial clinical and public health burden. According to the WHO Global Tuberculosis Report 2025, an estimated 10.7 million people developed TB and 1.23 million died from the disease in 2024, confirming that TB remains the world's leading cause of death from a single infectious agent (1). Beyond acute mortality, the burden of pulmonary TB extends into chronic respiratory morbidity, impaired physical functioning, and long-term reductions in quality of life, even among patients who complete anti-tuberculosis therapy successfully (2, 3). These observations have shifted attention from short-term microbiological cure to the broader concept of TB-associated respiratory disability and post-TB lung disease (1–3).

Over the past decade, increasing evidence has shown that pulmonary function impairment after TB is common and heterogeneous (2–5). Systematic reviews have demonstrated that TB survivors may develop obstructive, restrictive, or mixed ventilatory defects, and many also exhibit impaired gas exchange. In a recent meta-analysis, prior pulmonary TB was associated with significantly reduced lung function compared with controls, with FEV1 often more affected than FVC, suggesting a mixed pattern with a predominance of airflow limitation (3). Another systematic review of spirometry data from 14,621 individuals further confirmed that abnormal spirometry remains frequent after TB and that severe impairment is not uncommon (4). Longitudinal studies have also suggested that lung function abnormalities may evolve over time, with some patients transitioning from restrictive to obstructive phenotypes during treatment and follow-up (5). More recently, prospective data in adolescents indicated that impaired lung function may persist even after treatment completion, highlighting that TB-related respiratory sequelae may begin early and are not confined to older adults or patients with advanced structural lung destruction (2–5).

Despite this progress, important gaps remain. First, much of the existing literature has focused on post-treatment or post-TB lung disease, whereas pulmonary function patterns in patients with active pulmonary TB complicated by pulmonary infection are less well characterized. This distinction is clinically important because concurrent pulmonary infection may amplify airway inflammation, parenchymal injury, gas-exchange abnormalities, and short-term respiratory functional decline. Second, although TB coinfections with bacterial, fungal, and viral pathogens are increasingly recognized as contributors to disease severity and poorer outcomes, most available studies have concentrated on microbiological epidemiology, treatment response, or mortality rather than detailed pulmonary function phenotyping. Third, advances in bronchoalveolar lavage fluid metagenomic next-generation sequencing have improved pathogen detection in pulmonary infections, especially in complex or mixed infections, yet the relationship between clinically characterized pulmonary dysfunction and the broader infectious milieu remains insufficiently explored. Consequently, the clinical correlates of different pulmonary function impairment patterns in this subgroup of patients remain unclear (2, 3, 6–8).

Therefore, the present study aimed to characterize the patterns of pulmonary function impairment in patients with pulmonary TB complicated by pulmonary infection and to investigate their clinical correlates in a single-center retrospective cohort. By describing the distribution of ventilatory, diffusion, and respiratory reserve abnormalities and examining their associations with demographic, inflammatory, immunologic, and gas-exchange variables, we sought to provide a more clinically relevant understanding of respiratory functional heterogeneity in this high-risk population and to generate evidence that may support earlier recognition and more individualized management of TB-related pulmonary dysfunction (2–8).

## Materials and methods

### Study design and reporting standard

This single-center retrospective cross-sectional study based on hospitalization-phase data derived from an ongoing prospective clinical database and was conducted and reported in accordance with the Strengthening the Reporting of Observational Studies in Epidemiology (STROBE) statement (9).

### Study setting and participants

We retrospectively reviewed consecutive hospitalized patients with pulmonary TB complicated by pulmonary infection who were admitted to the Fourth People's Hospital of Ningxia Hui Autonomous Region between November 2024 and October 2025. Eligible patients were identified from the institutional clinical database established for pathogen detection using bronchoalveolar lavage fluid mNGS and related clinical evaluations. Although the analyzed variables were obtained during hospitalization, the present study was retrospective because all data were extracted from existing medical records and database entries after routine clinical care had already occurred. Although the parent project included prospective follow-up, the present analysis was restricted to hospitalization-phase data and did not include follow-up variables or longitudinal outcomes. The study sample was defined by the number of consecutive eligible patients during the study period who also had interpretable pulmonary function results available in the database.

Patients were included if they met all of the following criteria: (1) Diagnosis of pulmonary TB established on the basis of clinical, radiologic, microbiologic, and/or molecular evidence recorded in the medical chart; (2) Concurrent pulmonary infection judged by the treating physicians based on compatible clinical manifestations, imaging findings, and microbiological assessment; (3) Completion of bronchoalveolar lavage fluid mNGS during hospitalization; and (4) Availability of pulmonary function testing with interpretable results. Patients were excluded if they had: (1) Missing key pulmonary function data; (2) Pulmonary function maneuvers judged to be technically unacceptable or uninterpretable; (3) Insufficient core clinical or laboratory data for the planned analyses; or (4) Repeated admissions, in which case only the first eligible hospitalization was retained.

This study was approved by the Ethics Committee of the Fourth People's Hospital of Ningxia Hui Autonomous Region (approval number.2024-QSY-017). Given the retrospective nature of the study and the use of de-identified data, the requirement for written informed consent was waived by the ethics committee, if applicable according to local policy. The study was conducted in accordance with the Declaration of Helsinki and institutional requirements for retrospective clinical research.

### Data collection

Clinical data were extracted from the electronic medical record system and the institutional database using a standardized form. The collected variables included demographic characteristics, anthropometric data, presenting symptoms, time from symptom onset to diagnosis, relevant comorbidities, smoking history, routine laboratory results, inflammatory biomarkers, immune cell subsets, arterial blood gas parameters, oxygenation indices, microbiological findings, and pulmonary function parameters. Although chest imaging was reviewed in routine clinical care, detailed radiologic severity variables, including standardized CT severity scores, cavitary burden, lobar involvement, fibrosis, and bronchiectasis, were not systematically captured in the study database and were therefore not included in the present analysis. Smoking history was retrieved from routine clinical records as documented at admission and was used as a baseline clinical characteristic in the revised analysis. For the present study, only variables recorded during hospitalization were used, and no follow-up pulmonary function measurements or longitudinal outcome data were included in the analysis.

The laboratory variables included, when available, white blood cell (WBC) count, neutrophil percentage, lymphocyte count, C-reactive protein (CRP), procalcitonin (PCT), ESR, interleukin-6 (IL-6), albumin, and other routine biochemical indicators. Immune-related variables included CD4, CD8, NK cells, and Treg. Gas-exchange variables included PaO_2_, PaCO_2_, lactate, and oxygenation index.

### Pulmonary function testing and interpretation

Pulmonary function tests were performed during hospitalization according to routine clinical practice at our institution. Spirometry was conducted in accordance with the current American Thoracic Society/European Respiratory Society (ATS/ERS) technical standards for spirometry, and diffusion testing was performed according to the ERS/ATS standards for single-breath carbon monoxide uptake (10, 11). Interpretation of lung function patterns followed the contemporary ERS/ATS interpretive strategies for routine lung function tests (12).

The pulmonary function variables retrieved from the database included FVC, FEV1, FEV1/FVC, PEF, MEF75, MEF50, MEF25, MVV, TLC, RV, DLCO, and DLCO/VA, together with the physician-reported overall interpretation recorded in the pulmonary function report. Although raw pulmonary function measurements were available, pulmonary function phenotypes were not reclassified *post hoc* from raw numerical data or flow-volume curves using a centralized ATS/ERS-based algorithm. Instead, phenotype assignment was based on the final interpretation documented in the original clinical pulmonary function report. Each patient was categorized as having one or more of the following functional abnormalities: obstructive ventilatory impairment, restrictive ventilatory impairment, mixed ventilatory impairment, small airway dysfunction, diffusion impairment, and reduced respiratory reserve. These categories were not mutually exclusive. For the main comparative analyses, two prespecified binary outcome groups were constructed: with versus without diffusion impairment, and with versus without reduced respiratory reserve.

### Microbiological assessment and mNGS

Bronchoalveolar lavage fluid specimens were obtained during bronchoscopy as part of routine clinical evaluation. Conventional microbiological tests were performed according to institutional protocols, including smear, culture, and other pathogen-specific assays when clinically indicated. Bronchoalveolar lavage fluid mNGS was used for broad pathogen detection to assist in identifying bacterial, fungal, and viral organisms in patients with suspected complex pulmonary infection, consistent with current clinical applications of mNGS in lower respiratory tract infections (7, 8).

For the present study, the mNGS results were summarized at two levels. First, pathogens were classified into major categories, including bacteria, fungi, and viruses. Second, combined pathogen patterns were defined according to the coexistence of more than one pathogen category in the same patient. For the descriptive analysis, representative organisms with sufficient frequency in the cohort were also summarized individually. Because mNGS findings may reflect infection, colonization, or viral reactivation depending on the clinical context, all microbiological results were interpreted in conjunction with the contemporaneous clinical and radiologic information recorded in the medical chart.

### Outcome definition

The primary objective of this study was to characterize pulmonary function impairment patterns and their clinical correlates. Accordingly, the main outcomes were pulmonary function phenotypes extracted from the original clinical pulmonary function report. Because phenotype assignment was based on the final report interpretation rather than centralized *post hoc* recalculation from raw pulmonary function data, the present study was designed primarily to evaluate clinically documented functional patterns in real-world practice. Diffusion impairment was selected as the primary pulmonary function phenotype for the main analysis because it was frequent in this cohort and clinically relevant to parenchymal and gas-exchange abnormalities in pulmonary TB complicated by pulmonary infection. Reduced respiratory reserve was analyzed as a secondary major phenotype using the same binary grouping approach. The aim of this study was cross-sectional clinical characterization rather than longitudinal prognosis evaluation.

### Covariates

Potential clinical correlates were selected *a priori* on the basis of clinical plausibility and data availability. These included age, sex, body mass index (BMI), time from symptom onset to diagnosis, comorbid chronic airway disease, smoking history, inflammatory markers, immune status indicators, and gas-exchange parameters. To reduce multicollinearity in multivariable analyses, variables representing overlapping biological domains were not entered simultaneously in the same model. For example, CD4 and lymphocyte count were considered alternative indicators of immune status, whereas CRP and IL-6 were considered alternative indicators of systemic inflammatory burden; similarly, PaO_2_ and oxygenation index were treated as alternative indicators of oxygenation status. Smoking history was considered an important potential confounder in the interpretation of obstructive ventilatory abnormalities, diffusion impairment, and reduced respiratory reserve.

### Statistical analysis

Continuous variables were assessed for distributional characteristics before analysis. Normally distributed variables are presented as mean ± standard deviation and were compared using the Student's *t* test. Non-normally distributed variables are presented as median (interquartile range) and were compared using the Mann–Whitney *U* test. Categorical variables are presented as number (percentage) and were compared using the chi-square test or Fisher's exact test, as appropriate.

We first described the overall distribution of pulmonary function phenotypes in the study population. We then compared demographic, inflammatory, immunologic, microbiological, and gas-exchange characteristics between patients with and without specific pulmonary function abnormalities. Exploratory multivariable logistic regression analyses were subsequently performed to identify adjusted clinical associations of major pulmonary function phenotypes. Odds ratios and 95% confidence intervals were calculated.

The primary multivariable model used diffusion impairment as the dependent variable. Secondary models were constructed for reduced respiratory reserve and other major pulmonary function phenotypes when the number of events was sufficient to support stable estimation. Candidate covariates were selected based on clinical relevance and univariable screening, with careful attention to model parsimony given the sample size. To minimize overfitting, the number of variables entered into each final model was restricted according to the number of outcome events. Collinearity was assessed before model fitting, and only one representative variable from each highly correlated domain was retained in the final model. Because of the modest sample size, the multivariable models were intended as exploratory adjustment analyses rather than formal prediction models, and the adjusted estimates were interpreted as hypothesis-generating. No formal *a priori* sample size calculation was performed, because this study was a retrospective cross-sectional study of an existing clinical dataset and the final sample size depended on the number of consecutive eligible patients with interpretable pulmonary function data during the study period.

All statistical tests were two-sided, and a *P* value < 0.05 was considered statistically significant. Statistical analyses were performed using SPSS 27.0.

## Results

### Study population and baseline characteristics

A total of 72 hospitalized patients with pulmonary TB complicated by pulmonary infection who had interpretable pulmonary function results were included in the final analysis. The median age was 62.00 years (IQR, 53.75–72.25), and 42 patients (58.3%) were male. The mean BMI was 21.56 ± 3.23 kg/m^2^. Smoking history was documented in 25 patients (34.7%). The most common presenting symptoms were cough (95.8%) and sputum production (91.7%), followed by fatigue (58.3%), fever (26.4%), and night sweats (23.6%). Only 1 patient (1.4%) had a documented history of chronic obstructive pulmonary disease (COPD). The mean time to clinical improvement was 6.26 ± 2.96 days.

With regard to laboratory and physiologic variables, the median CD4 count on day 1 was 611.00 cells/μL (IQR, 423.50–712.50), the median WBC was 5.54 × 10^9^/L (IQR, 4.56–7.25), and the median lymphocyte count was 1.33 × 10^9^/L (IQR, 0.97–1.77). The median CRP was 8.25 mg/L (IQR, 1.53–43.77), and the median IL-6 was 7.35 pg/ml (IQR, 2.92–21.36). The median PaO2 was 72.00 mmHg (IQR, 63.00–77.00), and the mean oxygenation index was 327.86 ± 51.92 mmHg. Detailed baseline characteristics are shown in Table 1.

**Table 1:** Baseline characteristics of the study population.

Variable	Overall (*N* = 72)	Available *n*
Age, years	62.00 (53.75–72.25)	72
Male sex, *n* (%)	42 (58.3)	72
BMI, kg/m^2^	21.56 ± 3.23	72
Smoking history, *n* (%)	25 (34.7)	72
Cough, *n* (%)	69 (95.8)	72
Sputum production, *n* (%)	66 (91.7)	72
Fatigue, *n* (%)	42 (58.3)	72
Night sweats, *n* (%)	17 (23.6)	72
Fever, *n* (%)	19 (26.4)	72
History of COPD, *n* (%)	1 (1.4)	72
Time to clinical improvement, days	6.26 ± 2.96	72
CD4 count on day 1, cells/μl	611.00 (423.50–712.50)	72
WBC on day 1, × 10^9^/L	5.54 (4.56–7.25)	72
Neutrophil percentage on day 1, %	65.85 ± 12.07	71
Lymphocyte count on day 1, × 10^9^/L	1.33 (0.97–1.77)	72
CRP on day 1, mg/L	8.25 (1.53–43.77)	61
Albumin on day 1, g/L	38.05 ± 5.35	71
PaO_2_ on day 1, mmHg	72.00 (63.00–77.00)	65
Oxygenation index on day 1, mmHg	327.86 ± 51.92	65
IL-6 on day 1, pg/ml	7.35 (2.92–21.36)	67
PCT on day 1, ng/ml	0.05 (0.04–0.09)	67
FVC, L	3.01 ± 0.84	72
FEV1, L	2.47 ± 0.65	72
FEV1/FVC, %	84.39 ± 9.51	72
TLC-SB, L	5.47 ± 0.88	72
DLCO SB	8.06 ± 1.24	71
DLCO/VA	1.53 ± 0.21	72
MVV, L/min	92.97 ± 22.30	72

### Distribution of pulmonary function impairment patterns

Pulmonary function abnormalities were common in this cohort. The most frequent phenotype was diffusion impairment, observed in 37 of 72 patients (51.4%), followed by reduced respiratory reserve in 36 patients (50.0%) and obstructive ventilatory impairment in 30 patients (41.7%). Less common abnormalities included restrictive ventilatory impairment (16.7%), small airway dysfunction (13.9%), and mixed ventilatory impairment (13.9%). The distribution of pulmonary function impairment patterns is summarized in Table 2.

**Table 2:** Distribution of pulmonary function impairment patterns.

Pulmonary function impairment pattern	*n* (%)
Obstructive ventilatory impairment	30 (41.7)
Restrictive ventilatory impairment	12 (16.7)
Mixed ventilatory impairment	10 (13.9)
Small airway dysfunction	10 (13.9)
Diffusion impairment	37 (51.4)
Reduced respiratory reserve	36 (50.0)

Pulmonary function impairment patterns were extracted from the final interpretation documented in the original clinical pulmonary function report rather than from centralized post hoc ATS/ERS reclassification of raw numerical data. Categories may overlap; therefore, percentages do not sum to 100%.

### Comparison according to diffusion impairment status

When patients were stratified by diffusion impairment status, those with diffusion impairment were significantly more likely to be male than those without diffusion impairment (73.0% vs. 42.9%, *P* = 0.019). Smoking history showed a borderline between-group difference, with a higher proportion of smokers in the diffusion impairment group than in the non-diffusion impairment group (45.9% vs. 22.9%, *P* = 0.070). No significant between-group differences were observed for age, BMI, major respiratory symptoms, time to clinical improvement, CD4 count, WBC, neutrophil percentage, albumin, PaO_2_, oxygenation index, or IL-6 (all *P* > 0.05).

There was also a trend toward a higher lymphocyte count in patients with diffusion impairment compared with those without diffusion impairment [1.49 (1.05–1.82) vs. 1.12 (0.90–1.64) × 10^9^/L, *P* = 0.087]. In addition, CRP tended to be higher in the diffusion impairment group [12.66 (2.64–66.16) vs. 3.16 (1.44–29.01) mg/L, *P* = 0.166], although this difference did not reach statistical significance. PCT remained significantly higher in patients with diffusion impairment [0.06 (0.05–0.15) vs. 0.04 (0.03–0.08) ng/ml, *P* = 0.009]. Full comparisons are presented in Table 3.

**Table 3:** Comparison between patients with and without diffusion impairment.

Variable	Without diffusion impairment (*n* = 35)	With diffusion impairment (*n* = 37)	*P*-value
Age, years	62.00 (56.50–71.00)	62.00 (43.00–73.00)	0.484
Male sex, *n* (%)	15 (42.9)	27 (73.0)	0.019
BMI, kg/m^2^	21.73 ± 3.20	21.41 ± 3.30	0.677
Smoking history, *n* (%)	8 (22.9)	17 (45.9)	0.070
Cough, *n* (%)	34 (97.1)	35 (94.6)	1.000
Sputum production, *n* (%)	32 (91.4)	34 (91.9)	1.000
Fatigue, *n* (%)	20 (57.1)	22 (59.5)	1.000
Night sweats, *n* (%)	9 (25.7)	8 (21.6)	0.896
Fever, *n* (%)	9 (25.7)	10 (27.0)	1.000
History of COPD, *n* (%)	0 (0.0)	1 (2.7)	1.000
Time to clinical improvement, days	6.34 ± 2.80	6.19 ± 3.13	0.827
CD4 count on day 1, cells/μl	604.00 (414.50–780.00)	616.00 (437.00–692.00)	0.744
WBC on day 1, × 10^9^/L	5.30 (4.08–7.03)	5.64 (5.17–7.53)	0.171
Neutrophil percentage on day 1, %	65.43 ± 12.07	66.24 ± 12.22	0.780
Lymphocyte count on day 1, × 10^9^/L	1.12 (0.90–1.64)	1.49 (1.05–1.82)	0.087
CRP on day 1, mg/L	3.16 (1.44–29.01)	12.66 (2.64–66.16)	0.166
Albumin on day 1, g/L	37.34 ± 4.98	38.74 ± 5.67	0.275
PaO2 on day 1, mmHg	71.00 (62.00–79.00)	72.50 (64.50–75.00)	0.870
Oxygenation index on day 1, mmHg	325.54 ± 54.45	330.26 ± 49.93	0.717
IL-6 on day 1, pg/ml	7.54 (3.90–20.28)	7.35 (2.44–26.66)	0.960
PCT on day 1, ng/ml	0.04 (0.03–0.08)	0.06 (0.05–0.15)	0.009

### Comparison according to reduced respiratory reserve status

Patients were also grouped according to the presence or absence of reduced respiratory reserve. Most baseline demographic and clinical variables were comparable between the two groups, including age, sex, BMI, symptoms, time to clinical improvement, CD4 count, WBC, neutrophil percentage, lymphocyte count, CRP, albumin, PaO2, oxygenation index, smoking history, and PCT (all *P* > 0.05). However, IL-6 levels were significantly higher in patients with reduced respiratory reserve than in those without reduced respiratory reserve [13.18 (5.52–38.00) vs. 4.53 (1.94–12.28) pg/ml, *P* = 0.005]. This finding suggests a possible link between systemic inflammatory burden and impaired respiratory reserve in this population (Table 4).

**Table 4:** Comparison between patients with and without reduced respiratory reserve.

Variable	Without reduced respiratory reserve (*n* = 36)	With reduced respiratory reserve (*n* = 36)	*P*-value
Age, years	64.00 (48.25–72.25)	62.00 (55.75–72.25)	0.826
Male sex, *n* (%)	22 (61.1)	20 (55.6)	0.811
BMI, kg/m^2^	21.85 ± 3.39	21.28 ± 3.09	0.461
Smoking history, *n* (%)	13 (36.1)	12 (33.3)	1.000
Cough, *n* (%)	35 (97.2)	34 (94.4)	1.000
Sputum production, *n* (%)	33 (91.7)	33 (91.7)	1.000
Fatigue, *n* (%)	19 (52.8)	23 (63.9)	0.473
Night sweats, *n* (%)	8 (22.2)	9 (25.0)	1.000
Fever, *n* (%)	10 (27.8)	9 (25.0)	1.000
History of COPD, *n* (%)	0 (0.0)	1 (2.8)	1.000
Time to clinical improvement, days	6.33 ± 3.19	6.19 ± 2.74	0.844
CD4 count on day 1, cells/μl	611.00 (433.25–754.50)	611.00 (422.75–692.00)	0.951
WBC on day 1, × 10^9^/L	5.36 (4.12–7.79)	5.59 (4.94–6.95)	0.744
Neutrophil percentage on day 1, %	66.21 ± 12.69	65.50 ± 11.60	0.805
Lymphocyte count on day 1, × 10^9^/L	1.35 (1.06–1.77)	1.25 (0.94–1.75)	0.496
CRP on day 1, mg/L	5.54 (1.29–32.41)	9.16 (2.17–48.06)	0.373
Albumin on day 1, g/L	38.02 ± 5.16	38.07 ± 5.61	0.969
PaO2 on day 1, mmHg	73.00 (64.25–77.25)	69.00 (63.00–75.00)	0.486
Oxygenation index on day 1, mmHg	333.33 ± 51.18	322.56 ± 52.86	0.407
IL-6 on day 1, pg/ml	4.53 (1.94–12.28)	13.18 (5.52–38.00)	0.005
PCT on day 1, ng/ml	0.05 (0.03–0.07)	0.05 (0.04–0.10)	0.713

### Exploratory multivariable logistic regression analyses

In the exploratory multivariable logistic regression model for diffusion impairment, male sex, lymphocyte count, and smoking history were not independently associated with the outcome after adjustment. Likewise, a higher lymphocyte count showed a directionally positive but statistically non-significant estimate with diffusion impairment (adjusted OR 4.427, 95% CI 0.971–20.178, *P* = 0.055). Smoking history was not independently associated with diffusion impairment (adjusted OR 1.531, 95% CI 0.365–6.416, *P* = 0.560). Age, BMI, CRP, and oxygenation index were not independently associated with diffusion impairment (all *P* > 0.05).

In the exploratory multivariable model for reduced respiratory reserve, no variable reached statistical significance. Specifically, age, male sex, BMI, lymphocyte count, IL-6, and oxygenation index, and smoking history were not independently associated with reduced respiratory reserve after adjustment (all *P* > 0.05) (Table 5). These adjusted analyses should be interpreted cautiously because of the limited sample size and are intended primarily to assess the directional robustness of the observed associations rather than to establish definitive independent predictors.

**Table 5:** Exploratory multivariable logistic regression analyses of major pulmonary function phenotypes.

Outcome	Variable	Adjusted OR (95% CI)	*P*-value	Complete cases
Diffusion impairment	Age, years	1.022 (0.976–1.071)	0.351	56
Diffusion impairment	Male sex	3.461 (0.935–12.809)	0.063	56
Diffusion impairment	BMI, kg/m^2^	0.862 (0.690–1.076)	0.190	56
Diffusion impairment	Smoking history	1.531 (0.365–6.416)	0.560	56
Diffusion impairment	Lymphocyte count on day 1, × 10^9^/L	4.427 (0.971–20.178)	0.055	56
Diffusion impairment	CRP on day 1, mg/L	1.012 (0.995–1.028)	0.161	56
Diffusion impairment	Oxygenation index on day 1, mmHg	1.009 (0.994–1.024)	0.225	56
Reduced respiratory reserve	Age, years	0.991 (0.948–1.036)	0.683	62
Reduced respiratory reserve	Male sex	0.581 (0.179–1.889)	0.367	62
Reduced respiratory reserve	BMI, kg/m^2^	0.947 (0.795–1.128)	0.540	62
	Smoking history	1.782 (0.429–7.406)	0.427	62
Reduced respiratory reserve	Lymphocyte count on day 1, × 10^9^/L	0.788 (0.510–1.219)	0.285	62
Reduced respiratory reserve	IL-6 on day 1, pg/ml	1.017 (0.991–1.042)	0.197	62
Reduced respiratory reserve	Oxygenation index on day 1, mmHg	0.998 (0.988–1.009)	0.774	62

## Discussions

This study aimed to characterize pulmonary function impairment patterns in patients with pulmonary TB complicated by pulmonary infection and to explore their clinical correlates in a hospitalized real-world cohort. Three main findings emerged. First, pulmonary function abnormalities were common, with diffusion impairment and reduced respiratory reserve being the two most frequent phenotypes, followed by obstructive ventilatory impairment. Second, different functional phenotypes showed different clinical associations: diffusion impairment was more common in male patients in univariable analysis, whereas reduced respiratory reserve was associated with higher IL-6 levels. Third, in exploratory multivariable analysis, no variable was independently associated with diffusion impairment or reduced respiratory reserve after adjustment, although lymphocyte count showed a non-significant positive trend for diffusion impairment. with diffusion impairment, while no independent correlate of reduced respiratory reserve was identified. Taken together, these findings suggest that pulmonary dysfunction in this setting is heterogeneous and cannot be fully explained by a single ventilatory or inflammatory mechanism. They also raise the possibility that diffusion impairment may be clinically underrecognized in active pulmonary TB complicated by pulmonary infection.

Previous studies have established that pulmonary function impairment is common in patients with pulmonary TB, but the predominant functional pattern remains variable across studies (3–5, 13). Systematic reviews and longitudinal cohorts have reported obstructive, restrictive, and mixed defects, with many studies emphasizing spirometric abnormalities and airflow limitation (3–5, 13). In contrast, our cohort of hospitalized patients with active pulmonary TB and concurrent pulmonary infection showed a predominance of diffusion impairment and reduced respiratory reserve. This difference may reflect the distinct clinical context of our study, in which ongoing inflammation, acute respiratory compromise, and more severe inpatient disease may have contributed more strongly to impaired gas transfer and ventilatory capacity than to classical ventilatory restriction alone (5, 13, 14). Because our analysis also extended beyond spirometric findings, non-ventilatory abnormalities may have been more readily identified. However, given the absence of standardized radiologic severity measures, the contribution of structural lung damage could not be directly assessed and should not be inferred from our data alone.

The clinical correlates of specific pulmonary function phenotypes in pulmonary TB remain incompletely defined (15–22). Previous studies suggest that sex, smoking exposure, and inflammatory burden may influence respiratory impairment, although most reports have examined overall lung dysfunction rather than phenotype-specific outcomes (15–22). In our study, diffusion impairment was more common in male patients in univariable analysis, whereas reduced respiratory reserve was associated with higher IL-6 levels. Previous studies have also linked male sex to poorer respiratory outcomes after pulmonary TB (15–17, 20–22) and IL-6 to TB-related lung impairment (18, 19). After incorporating smoking history into the multivariable model, male sex was no longer independently associated with diffusion impairment, suggesting that the apparent sex-related difference may have been partly explained by smoking or other correlated clinical factors. By contrast, IL-6 remained clinically relevant as a univariable marker of reduced respiratory reserve but did not retain independent significance after adjustment. These findings suggest that pulmonary dysfunction in this setting is not biologically uniform and that different functional phenotypes may reflect different combinations of host, exposure-related, and inflammatory influences.

The independent determinants of specific pulmonary function phenotypes in pulmonary TB remain uncertain, and previous studies have reported inconsistent results across different populations and functional endpoints. Prior work has shown that male sex is often associated with more severe TB presentation and poorer outcomes, whereas immune-related markers may also contribute to pulmonary injury severity (20–22). In our study, after incorporating smoking history into the analysis, male sex was not independently associated with diffusion impairment, while lymphocyte count showed a non-significant positive trend. This pattern suggests that diffusion impairment may be influenced by a combination of host-related biological factors and exposure-related confounding rather than by sex alone. By contrast, no independent correlate of reduced respiratory reserve was identified, indicating that this phenotype may reflect a more complex and multifactorial process not fully captured by the available clinical variables in a relatively small retrospective cohort. Given the limited sample size, these multivariable findings should be interpreted as exploratory rather than confirmatory.

This study has several strengths. It focused on a clinically relevant but relatively underexplored population of patients with active pulmonary TB complicated by pulmonary infection, and it evaluated pulmonary dysfunction beyond spirometric abnormalities alone by including diffusion impairment and reduced respiratory reserve. In addition, pulmonary function findings were interpreted together with inflammatory, immunologic, and gas-exchange variables, which enhances the clinical relevance of the analysis in a real-world hospitalized setting. These strengths should be considered alongside the following limitations.

Several limitations of this study should be acknowledged. First, this was a single-center retrospective cross-sectional study conducted in a specialized infectious disease hospital. As a result, the study population may not fully represent the broader spectrum of patients with pulmonary TB complicated by pulmonary infection, particularly those managed in general hospitals or outpatient settings. This may limit the generalizability of our findings and may also have introduced selection bias toward patients with more severe or clinically complex disease. Future multicenter prospective studies involving different levels of care and more diverse patient populations are needed to improve external validity. Second, the sample size was relatively modest, especially after restricting the analysis to patients with interpretable pulmonary function results. This limited the statistical power of the study and may have reduced our ability to detect independent associations in multivariable models, particularly for less frequent pulmonary function phenotypes. Some borderline findings should therefore be interpreted with caution. In particular, the multivariable logistic regression analyses were constrained by the limited number of complete cases and outcome events relative to the number of covariates. Although these models were intentionally kept parsimonious and used only as exploratory adjusted analyses, overfitting and instability of the adjusted estimates cannot be excluded. In addition, because the sample size was determined by the number of available eligible patients in an existing database rather than by a formal *a priori* calculation, the study should be interpreted as an observational exploratory cross-sectional study rather than a definitively powered association study. Third, because of the retrospective design, several potentially important variables were unavailable or incompletely recorded, including some factors that may influence pulmonary function interpretation, such as detailed smoking exposure metrics (e.g., pack-years, smoking duration, and former-versus-current smoking status), standardized radiologic severity measures, and standardized pulmonary function reference parameters. Although smoking history was added in the revised dataset, the available smoking information remained limited in granularity and therefore could not fully account for smoking-related confounding. In particular, the absence of systematically collected imaging variables, such as CT severity scores, cavitary disease, lobar involvement, fibrosis, and bronchiectasis, limited our ability to correlate pulmonary function abnormalities with structural lung damage and to distinguish functional impairment related to active inflammatory change from that related to chronic anatomic sequelae. This may have resulted in residual confounding and may have limited the precision of phenotype classification. Future prospective studies should incorporate more comprehensive clinical, imaging, and functional data collection to allow a more refined assessment of the determinants of pulmonary dysfunction. Fourth, although raw pulmonary function measurements were available in the database, phenotype assignment in this study was based on the final physician-reported interpretation in the original clinical pulmonary function report rather than on centralized reclassification of raw numerical data and flow-volume curves using uniform ATS/ERS criteria. This is an important methodological limitation. Because phenotype classification depended on routine clinical reporting, inter-reader variability and classification inconsistency cannot be excluded, particularly for categories such as diffusion impairment, small airway dysfunction, and reduced respiratory reserve. This limitation may have affected the precision, reproducibility, and comparability of phenotype definitions and therefore warrants caution when interpreting phenotype-specific associations. Future studies should incorporate standardized *post hoc* adjudication of complete pulmonary function datasets using prespecified ATS/ERS-based criteria. Fifth, although bronchoalveolar lavage fluid mNGS provided valuable microbiological information, the detected organisms may not always represent definite causative pathogens, as some findings may reflect colonization, contamination, or viral reactivation rather than true active infection. This issue may have influenced the interpretation of associations between pulmonary infection and functional impairment. Future studies should combine mNGS results with stricter clinical adjudication, quantitative microbial burden assessment, and longitudinal follow-up to better distinguish clinically relevant infection from incidental detection. Finally, the present study was cross-sectional in its functional assessment, and pulmonary function was evaluated during hospitalization rather than longitudinally across treatment and recovery. Therefore, we were unable to determine whether the observed abnormalities were transient, progressive, or persistent after clinical improvement. Future longitudinal studies with repeated pulmonary function assessments are needed to clarify the trajectory of diffusion impairment, ventilatory defects, and reduced respiratory reserve over time. Accordingly, the present findings should be interpreted as associations based on clinically reported pulmonary function phenotypes in routine practice rather than as definitive phenotype assignments derived from centralized physiological reclassification. Because pulmonary function variables were analyzed only within the hospitalization phase and no follow-up outcome was included, the present findings should be interpreted as cross-sectional associations rather than longitudinal relationships.

Despite these limitations, the present study still provides clinically relevant preliminary evidence that diffusion impairment may be underrecognized in patients with active pulmonary TB complicated by pulmonary infection. In our cohort, diffusion impairment was the most frequent pulmonary function phenotype, suggesting that functional abnormalities related to gas transfer may warrant greater attention in routine assessment of this patient group. Future studies should strengthen this line of investigation by incorporating standardized ATS/ERS-based reclassification of pulmonary function data, systematic radiologic correlation, clearer microbiologic and clinical definitions of concomitant pulmonary infection, and multicenter prospective designs with longitudinal follow-up to determine the persistence and clinical consequences of these functional abnormalities.

## Conclusion

Pulmonary function impairment was common in patients with pulmonary TB complicated by pulmonary infection, with diffusion impairment and reduced respiratory reserve as the predominant phenotypes. Theoretically, these findings support that pulmonary dysfunction in this population is heterogeneous and involves not only ventilatory defects but also impaired gas transfer and respiratory capacity. Clinically, comprehensive pulmonary function assessment may help improve functional evaluation, risk recognition, and individualized management for both patients and treating physicians. These findings may also help inform future studies on functional phenotyping and follow-up assessment in patients with pulmonary TB complicated by pulmonary infection.

## Data Availability

The original contributions presented in the study are included in the article/supplementary material, further inquiries can be directed to the corresponding author.
